# An unusual case of peripartum cardiomyopathy manifesting with multiple thrombo-embolic phenomena

**DOI:** 10.1186/1477-9560-5-18

**Published:** 2007-10-29

**Authors:** Uzoma N Ibebuogu, John W Thornton, Guy L Reed

**Affiliations:** 1 Division of Cardiology, Medical College of Georgia, Augusta, Georgia, USA

## Abstract

Peripartum cardiomyopathy (PPCM) is a rare form of heart failure with a reported incidence of 1 per 3000 to 1 per 4000 live births and a fatality rate of 20%–50%. Onset is usually between the last month of pregnancy and up to 5 months postpartum in previously healthy women. Although viral, autoimmune and idiopathic factors may be contributory, its etiology remains unknown. PPCM initially presents with signs and symptoms of congestive heart failure and rarely with thrombo-embolic complications. We report an unusual case of PPCM in a previously healthy postpartum woman who presented with an acute abdomen due to unrecognized thromboemboli of the abdominal organs. This case illustrates that abdominal pain in PPCM may not always result from hepatic congestion as previously reported, but may occur as a result of thromboemboli to abdominal organs. Further research is needed to determine the true incidence of thromboemboli in PPCM.

## Background

Peripartum cardiomyopathy (PPCM) is a rare form of heart failure of unknown cause with a reported incidence of 1 per 3000 to 1 per 4000 live births. Onset is usually between the last month of pregnancy and up to 5 months postpartum in previously healthy women, with a reported fatality rate of 20%–50%. Although viral, autoimmune and idiopathic factors may be contributory, its etiology remains unknown. PPCM usually presents initially with signs and symptoms of heart failure and rarely with thrombo-embolic complications. We report an unusual case of PPCM in a previously healthy woman who presented with an acute abdomen due to multiple thromboemboli.

## Case presentation

**Figure 1:**
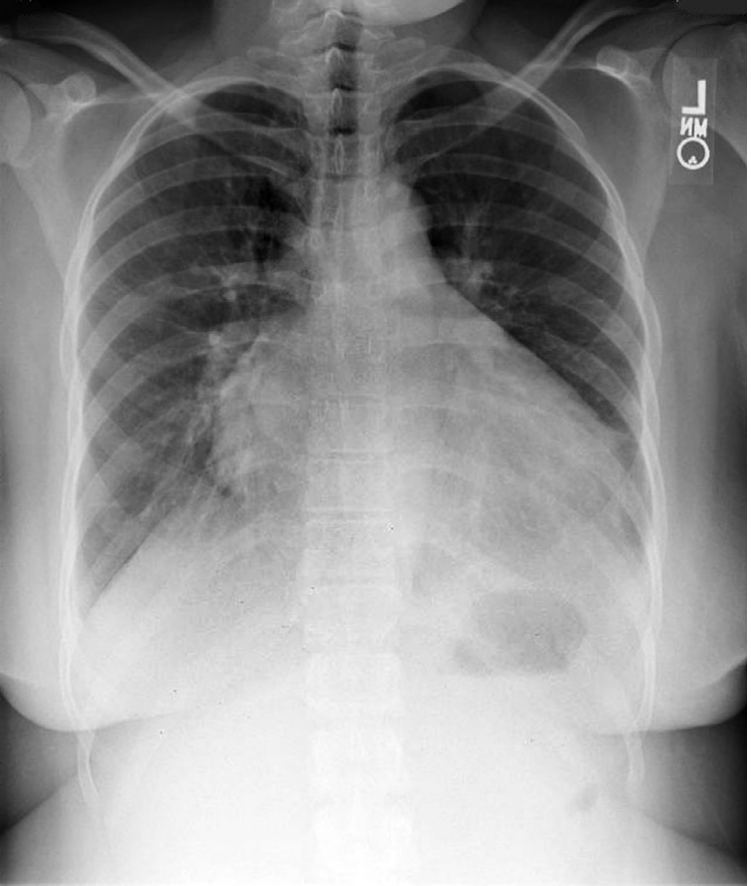
Chest x-ray showing moderate cardiomegaly and prominent bibasilar interstitial markings.

**Figure 2:**
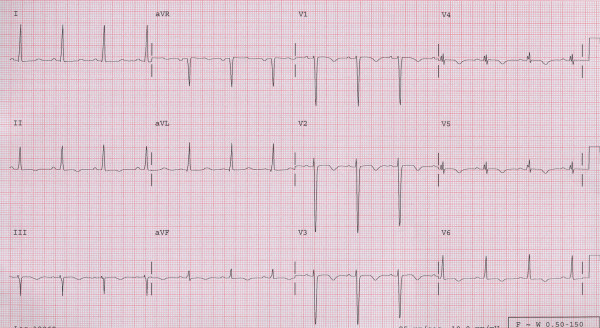
Electrocardiogram showing diffuse T wave inversions.

**Figure 3:**
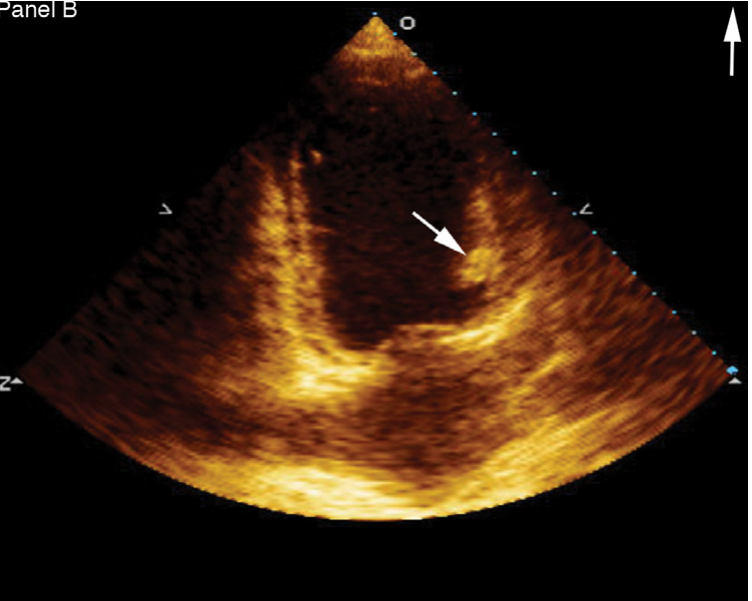
Transthoracic echocardiography showing left ventricular thrombus (arrow).

**Figure 4:**
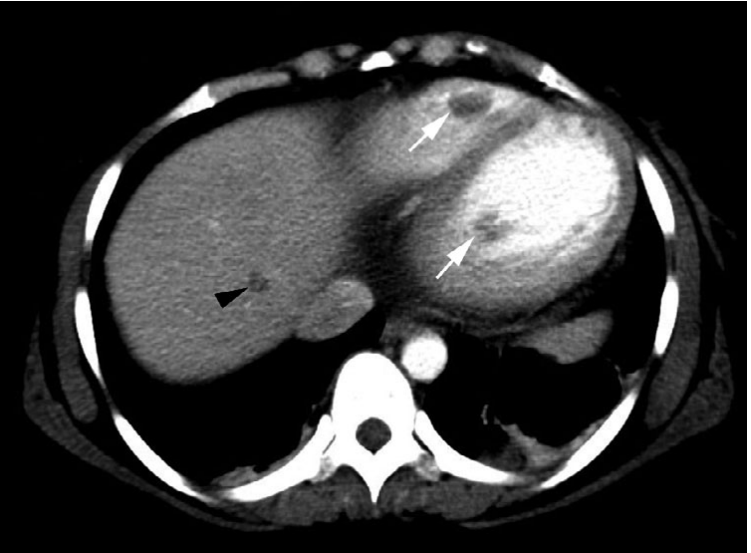
Computed tomography scan showing biventricular thrombi (white arrow) and a low attenuation lesion in the right lobe of the liver (black arrow).

**Figure 5:**
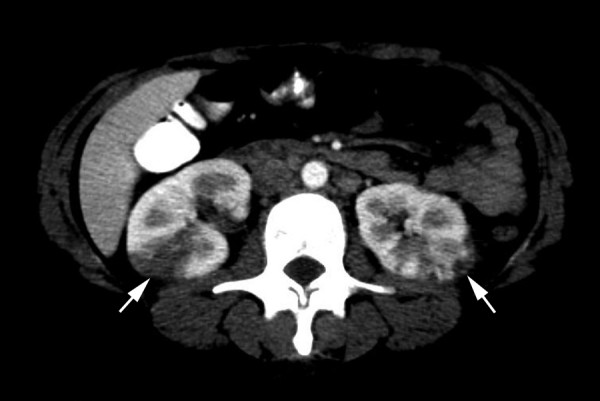
Abdominal computed tomography scan showing multiple wedge defects in both kidneys.

A 24 year old gravida 5 woman with no history of alcohol or drug abuse, and no previous history of cardiovascular disease, presented to the emergency department (ED) 5 months after an uneventful full term spontaneous vaginal delivery followed by tubal ligation. She complained of a severe epigastric and right upper quadrant abdominal pain, nausea and vomiting. Physical examination revealed normal vital signs, epigastric and right upper quadrant tenderness without peritoneal signs. Abdominal ultrasound showed thickened anterior gall bladder wall without stones or sludge and no pericholecystic fluid. Liver enzymes and amylase were within normal limits. She was given analgesics and subsequently discharged from the ED following complete resolution of her symptoms. Three days later she returned with a severe, worsening right upper quadrant and epigatric pain, nausea and shortness of breath. She also complained of left lower extremity and back pains. At this time, her blood pressure was 130/100 mmHg with a heart rate of 100. She had bilaterally decreased air entry on lung examination, intermittent apical third heart sound with a regular rate and rhythm, right upper quadrant and epigastric tenderness, bilateral costovertebral angle tenderness and a negative Murphy's sign. An acute abdominal x-ray series was normal. Chest x-ray (Fig. 1) showed moderate cardiomegaly and prominent bibasilar interstitial markings. Electrocardiography (Fig. 2) revealed a normal axis with diffuse T-wave inversion. A transthoracic echocardiography (TTE) (Fig. 3) revealed biventricular dilatation with a left ventricular end-diastolic dimension of 65 mm, a depressed fractional shortening of 9%, an ejection fraction of <15% and a left ventricular anterior wall thrombus. The patient was diagnosed with peripartum cardiomyopathy with congestive heart failure, liver congestion and a left ventricular mural thrombus. She was placed on bed rest with telemetry monitoring and started on therapeutic dose of low molecular weight heparin, nitrates, diuretics and an ACE inhibitor. Although, her congestive heart failure symptoms improved, her abdominal pain worsened. She had elevated lactic acid level (3.3 mmol/L) and white cell count (12,200/mm3), with no evidence of liver congestion on imaging. Right heart catheterization revealed normal cardiac output and right heart pressures with no evidence of intracardiac shunt, and a cardiac index of 2.6 L/min/m2. Computed tomography (CT) scan revealed findings suggestive of a biventricular thrombi and an 8 mm low attenuation lesion at the dome of the right lobe of the liver (Fig. 4), multiple wedge defects in both kidneys (Fig. 5), and a partially occlusive thrombus within the common iliac and right external iliac arteries. An arteriogram subsequently confirmed bilateral iliac and superficial femoral artery thrombi. Assays for IgG and IgM anticardiolipin antibodies were negative (6.8 GPL and <4.0 MPL respectively), and rheumatoid factor was less than 20 IU/mL. HIV I and II, hepatitis B and C, and RPR, were all negative and her ferritin level was within normal limits. She underwent successful bilateral lower extremity thrombectomies. Her renal function remained stable throughout her admission. She improved clinically on anticoagulation and heart failure therapy and repeat TTE 5 days after she presented showed no evidence of thrombi in either ventricle. Her lactic acid levels normalized on day 6 of hospitalization and she was discharged 2 weeks after she presented with follow up in the outpatient cardiology clinic.

## Discussion

Peripartum cardiomyopathy (PPCM) is a rare form of dilated cardiomyopathy that is associated with a high maternal morbidity and mortality, reportedly accounting for 4% of maternal deaths in the United States [1,2]. It is defined according to the following four criteria: 1) Development of cardiac failure in the last month of pregnancy or within five months of delivery; 2) absence of identifiable cause for the heart failure; 3) absence of recognizable heart disease prior to the last month of pregnancy; and 4) left ventricular systolic dysfunction demonstrated by echocardiographic criteria [1,3]. The exact etiology remains unknown. The incidence of PPCM ranges from 1:3000 to 1:4000 live births and it affects 1000 to 1300 women in the United States each year [4]. The mortality rate is reportedly 20% to 50%, and death occurs as a result of progressive heart failure, arrhythmias and thromboembolism [5]. PPCM presents initially with signs and symptoms of heart failure and rarely with thromboembolic complications [3,6-8]. The occurrence of thromboembolism in PPCM may be due to the hypercoagulable state of pregnancy and the left ventricular dysfunction which causes a relative blood stasis [8]. This warrants the initiation of anticoagulation therapy in the presence of severe left ventricular dysfunction. Upper abdominal discomfort occurs in approximately half of the patients diagnosed with PPCM as a result of an enlarged congested liver [9]. Our patient's initial presentation was with severe abdominal pain. After she was diagnosed with PPCM, her abdominal pain was initially ascribed to hepatic enlargement and congestion due to congestive heart failure. Due to worsening of her abdominal pain, despite resolution of her congestive heart failure symptoms, and normal right heart pressures on cardiac catheterization, a computed tomography contrast scan of the abdomen was ordered. This revealed multiple thromboemboli of the heart, kidneys, and common iliac and superficial femoral arteries. Based on the nature of her symptoms, and the occurrence of multiple emboli in the abdominal organs, ischemia from thromboemboli was the determined cause of her severe abdominal pain. Our patient was started on anticoagulation at the onset due to the presence of an intracardiac thrombus with severe systolic dysfunction on echocardiography. This played a role in the termination of the thromboembolic process and the resolution of her abdominal symptoms. The current consensus in the management of PPCM is to initiate anticoagulation therapy in the presence of severe left ventricular function (LVEF ≤ 35%) [1]. However, abdominal pain in the absence of congestive heart failure symptoms, may indicate the presence of thromboemboli of the abdominal organs. This may be a further indication for anticoagulation. Early diagnosis and treatment of PPCM are essential for a favorable outcome and poor prognostic factors include high parity, twin gestation, age greater than 30 years, and a late onset of symptoms after delivery [10]. Our patient is multiparous and her clinical presentation occurred 5 months postpartum. A high clinical index of suspicion, cardiomegaly and a severe left ventricular dysfunction prompted accurate diagnosis and a successful therapy. In patients with PPCM the return of the left ventricle size and function to normal in the first 5 months of the postpartum period is a good prognostic factor. This usually occurs in 50% of patients with PPCM. Our patient's left ventricular dysfunction persisted greater than 6 months after hospital discharge, and she is currently awaiting heart transplant.

## Conclusion

Patients with peripartum cardiomyopathy (PPCM) may rarely be predisposed to thromboembolic phenomenon due to blood stasis resulting from the hypercoagulable state of pregnancy and the depressed left ventricular systolic function typical of this disease. Abdominal pain may not always indicate the presence of hepatic enlargement and congestion as previously believed, but may occur as a result of non-occlusive thromboemboli of the bowel or other abdominal organs as reported in our patient. This may be a further indication for early initiation of anticoagulation to prevent a potential adverse outcome from bowel or other abdominal organ ischemia. Further research is needed to determine the true incidence of thromboemboli in PPCM.

## Abbreviations

PPCM – Peripartum Cardiomyopathy; CT – Computed tomography; ED – Emergency department.

## Competing interests

The author(s) declare that they have no competing interests.

## Authors' contributions

UI, JT and GR conceived of the study, tacked out responsibility for diagnosis of the patient, their evolution and elaborated in the design of this case and draft of the manuscript. UI also retrieved the images while JT edited the final text.

All authors read and approved the final manuscript.
